# A Marine Sulfate-Reducing Bacterium Producing Multiple Antibiotics: Biological and Chemical Investigation

**DOI:** 10.3390/md7030341

**Published:** 2009-07-21

**Authors:** Yi Zhang, Jun Mu, Xiaojie Gu, Chenyan Zhao, Xiaoliang Wang, Zeping Xie

**Affiliations:** Department of Biotechnology, School of Environmental and Chemical Engineering, Dalian Jiaotong University, 116028 Dalian, China; E-Mails:zhangyi@djtu.edu.cn (Y.Z.);guxiaojie2003@163.com (X.J.G);fdpeggy001@yahoo.com.cn (C.Y.Z.);xushikong@126.com (X.L.W.);xiezepingdalian@163.com (Z.P.X)

**Keywords:** marine sulfate-reducing bacterium, isolation, identification, broad-spectrum antimicrobial activity, mono-n-butyl phthalate

## Abstract

A marine sulfate-reducing bacterium SRB-22 was isolated by means of the agar shake dilution method and identified as *Desulfovibrio desulfuricans* by morphological, physiological and biochemical characteristics and 16S rDNA analysis. In the bioassay, its extract showed broad-spectrum antimicrobial activity using the paper disc agar diffusion method. This isolate showed a different antimicrobial profile than either ampicillin or nystatin and was found to produce at least eight antimicrobial components by bioautography. Suitable fermentation conditions for production of the active constituents were determined to be 28 day cultivation at 25 °C to 30 °C with a 10% inoculation ratio. Under these conditions, the SRB-22 was fermented, extracted and chemically investigated. So far an antimicrobial compound, mono-*n*-butyl phthalate, and an inactive compound, thymine, have been isolated and characterized.

## 1. Introduction

Obligate anaerobic microbes mainly include photosynthetic bacteria, sulfate-reducing bacteria, methanogenic bacteria, denitrifying bacteria and so on. Because of their unique enzyme systems and metabolic pathways, these microorganisms lead quite different lives from the common aerobic organisms and accordingly, they should theoretically produce structurally quite different secondary metabolites. However, our knowledge about their bioactive compounds, their potential biomedical use as drug lead compounds, and their chemecological role has been truly limited. This situation may be due to the difficulties in the isolation and cultivation of these anaerobic microorganisms. Nevertheless, some recent progress in these areas inspired us to continue probing into the anaerobic microbial world to investigate its natural products chemistry. For example, 3,7,12-trihydroxy-24-cholanic acid methylester, a potent antimicrobial agent, was reported to be produced by a facultative *Streptococcus faecium* when it was cultured under strict anaerobic conditions [1]. Also reported was the bacteriocin-like substance(s) produced by a strict anaerobic *Fusobacterium mortiferum* [2].

Sulfate-reducing bacteria are widely distributed in deep water and sediments of lakes, rivers, and oceans. With the capacity to use sulfate, sulfite, thiosulfate or even elemental sulfur as electron receptors instead of oxygen in their respiratory chain, they participate in the recycle of elemental sulfur in Nature. The search for bioactive compounds from this special microbial resource and their possible biomedical use is an interesting topic that has rarely been reported before. In our research, a marine sulfate-reducing bacterium SRB-22 was isolated from a sea water sample. Herein we report its isolation, identification, broad-spectrum antimicrobial activity, fermentation conditions, and preliminary chemical investigation of the compounds responsible for the observed antimicrobial properties.

## 2. Results and Discussion

### 2.1. Strain Isolation

The benthal sea water sample for strain isolation was collected in the Dalian sea area, China. Isolation was performed by an agar-shake-dilution purification process following an enrichment cultivation in sulfate-containing medium [3]. An anaerobic vibrio-like bacterium was obtained. This isolate was able to form black colonies within the solid Fe^2+^-containing medium and gave out the typical rotten egg odor of H_2_S in liquid culture, indicating that it was a sulfate-reducing bacterium. Accordingly it was designated as SRB-22.

The agar shake-dilution technique is a more convenient method than the usually used Hungate rolling tube technique [4]. In this method, the mixture of melted paraffin wax and paraffin oil (1:3, v/v) was poured onto the surface of an agar tube culture to insulate the medium from oxygen. Vitamin C and sodium thioglycolate were added to maintain a low redox potential in the medium. Under these anaerobic conditions, the anaerobic bacteria grew well and formed colonies.

### 2.2. Taxonomy

**Figure 1:**
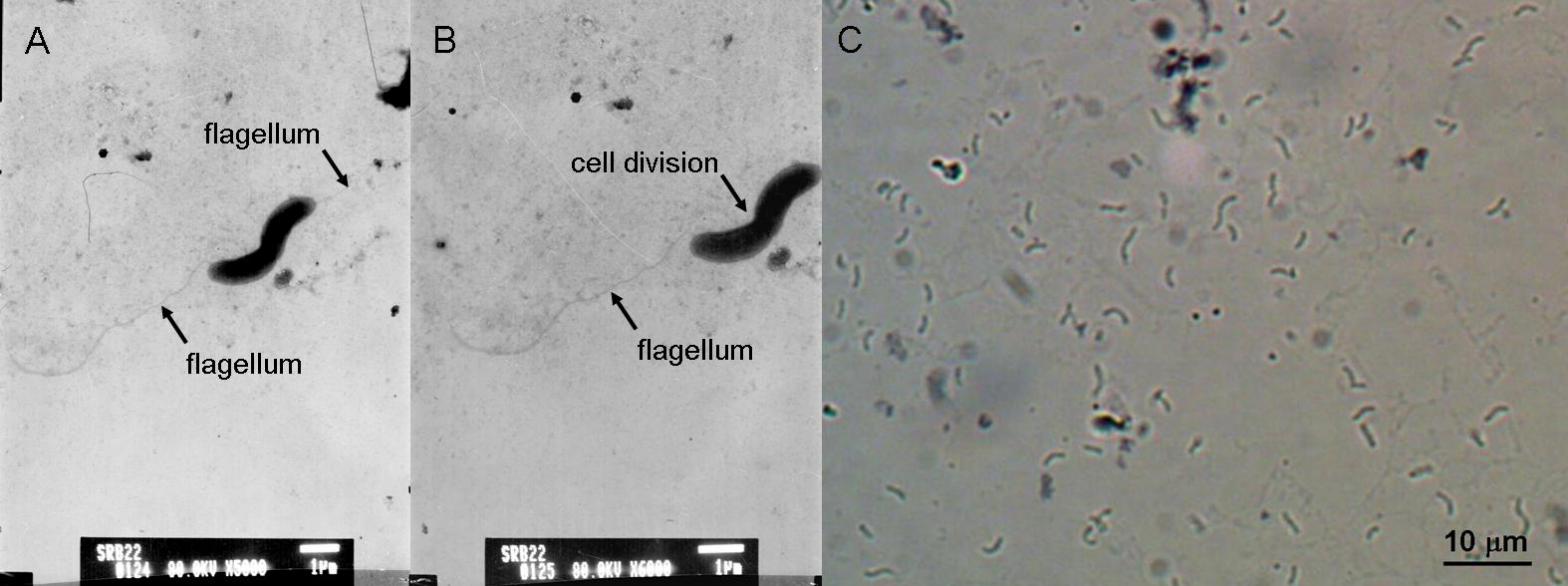
The Transmission Electronic Microscopic (A–B) and Light Microscopic (C) images of SRB-22. A. Showing two newly divided cells with single polar flagellum; B. Enlarged image of A.

The isolate SRB-22 was a Gram negative bacterium. Its cells were curved, with a width of 0.6–0.8 μm and a length of 2.0–5.0 μm, having a single polar flagellum for movement (Figure 1 A–C). This isolate was an obligate anaerobe. As mentioned above, it was able to produce H_2_S and FeS in media containing sulfate and Fe^2+^. The desulfoviridin test gave a positive result, revealing that desulfoviridin, a characteristic sulfites reductase for the most species of genus *Desulfovibrio*, was present in the cells of SRB-22 [5].

In the physiological and biochemical experiment, the SRB-22 isolate showed the ability to use lactate, pyruvate, malate, and choline, but not acetate or butyrate as electron donors in the presence of sulfate as an electron acceptor. Also it was able to grow fermentatively on malate and pyruvate when lacking sulfate as electron acceptor. Growth factors, i.e. yeast extract or multiple vitamins, were not necessary for its growth.

The above morphological, physiological and biochemical characteristics showed that the isolate SRB-22 was very similar to the *D. desulfuricans* sp. recorded in Bergery’s Manual of Systematic Bacteriology [5]. The PCR amplification of 16S rDNA from isolate SRB-22 gave a sequence of 1,467 bp in length, which was submitted and deposited in GenBank with the accession number of FJ873799. Based on a BLASTn search of GenBank, the closest matches to isolate SRB-22 were *Desulfovibrio desulfuricans* subsp. *desulfuricans* AF192153 (nucleotide identity: 100%), *D. desulfuricans* M34113 (nucleotide identity: 100%) and *D. desulfuricans* DQ092636 (nucleotide identity: 99%). On the basis of the above morphological, physiological and biochemical characteristics, and 16S rDNA analysis, the isolate SRB-22 was finally identified as *Desulfovibrio desulfuricans*.

### 2.3. Antimicrobial Activity

In an antimicrobial test using the paper disk diffusion method, the organic extract of the isolate SRB-22’s ferment broth was screened on its activity against five test bacteria and three test fungi (yeasts). An autoclaved blank medium extract was used as a blank control. The dose of concentrated extract on each paper disc was equal to the amount of substances extractable from 10 mL of ferment broth.

**Figure 2:**
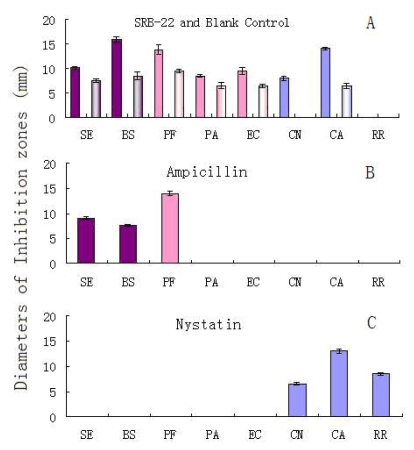
The antimicrobial activities of the organic extract of SRB-22 isolate, blank control, ampicillin at 6 μg/mL, and nystatin at 100 μg/mL. Abbreviations: SE = *Staphylococcus epidermidis*, BS = *Bacillus subtilis*, PF = *Pseudomonas fluorescens*, PA = *P. aeruginosa*, EC = *Escherichia coli*, CN = *Cryptococcus neoformans*, CA = *Candida albicans*, RR = *Rhodotorula rubra*. The respective light color columns in A show the activities of blank control, i.e., the extract of blank medium.

The results are shown in Figure 2A. After adjustment for the effect of blank medium, the SRB-22 extract showed solid inhibitory activity against both of the G^+^ bacteria (*Staphylococcus epidermidis* and *Bacillus subtilis*), all the three G^−^ bacteria (*Pseudomonas fluorescens*, *P. aeruginosa* and *Escherichia coli*), and two of the three fungi (*Cryptococcus neoformans* and *Candida albicans*). It was noticed that the blank control also showed moderate activity against five bacteria and one fungus, which could have been caused by residual vitamin C still present after autoclaving. As is known, vitamin C is reported to possess antimicrobial activities [6–7]; however, it can be largely destroyed by Fe^2+^ in the medium and the high temperatures during the autoclaving process [8–9].

Since the antimicrobial spectrum of the SRB-22 extract was different from that of the ampicillin and nystatin positive controls (Figure 2B and 2C), this isolate was presumed to be able to produce antimicrobial agents with different mechanisms of action than the positive controls.

**Figure 3:**
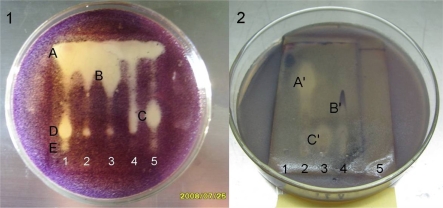
The antimicrobial bioautographies of fractions of SRB-22 extract against *Bacillus subtilis* (1) and *Candida albicans* (2).

To reveal the antimicrobial constituents clearly by bioautography, 20 mg of SRB-22 extract was partitioned into five fractions (fractions 1–5) by preparative thin layer chromatography (pTLC). The anti-*Bacillus subtilis* bioautography of these fractions clearly showed that there were at least five antibacterial components A–E with quite different *Rf* values (Figure 3-1).

The remarkable differences in their polarity suggested different structure types, so it is possible that the SRB-22 isolate could produce various different types of antibiotics. As to their mechanisms of action, they could be either different types of antibiotics with broad-spectrum activity towards G^+^ and G^−^ bacteria, or ones with specific killing mechanisms towards different types of bacteria. Likewise, the anti-*C. albicans* bioautography of the fractions also showed the presence of at least three active components A′–C′ with remarkably different polarities (Figure 3-2). The structures and pharmacology of the active compounds is under investigation and will be reported in due course.

Furthermore, according to their distribution in the fractions and *Rf* values, most of the antibacterial components should be different from the antifungal ones. For example, spots A, D and E in fraction 1 showed anti-*B. subtilis* activity while no anti-*C. albicans* activity, as did spot B in fraction 3 and C in fraction 5. Likewise, antifungal spot A′ in fraction 2 and spot C′ in fraction 3 didn’t show remarkable antibacterial activity.

As is known, vitamin C has antimicrobial activity and strong polarity. When analyzed together with the SRB-22 extract under the same TLC condition (CH_2_Cl_2_/MeOH/triethylamine, v/v/v, 10:1:0.02), pure vitamin C showed a very low *Rf* value of about 0–0.15. After autoclaving and possible metabolism, part of the vitamin C could possibly still remain in the culture medium and thus be mixed into the final organic extract. Fraction 5 was the most polar one obtained from the extract, so the residue of vitamin C was probably contained therein. Nevertheless, this fraction was eluted from the silica from the most polar band on the pTLC plate by a medium polarity solvent mixture, CH_2_Cl_2_/MeOH (v/v, 1:1), and was also finally dissolved in this solvent, so accordingly, the content of vitamin C in this sample should be limited. In view of similar polarities, the weak inhibitory spot with the *Rf* value 0.1–0.2 in fraction 5 was speculated to be due to the residue of vitamin C. Similarly, the other remaining strong polar organic salts contained in medium, including sodium thioglycolate and sodium lactate, should also be not the main active components in the bioautographies.

### 2.4. Study on Fermentation Conditions

For further chemical investigation of the antimicrobial compounds, enough active crude extract was necessary. To obtain high yield of the active constituents, different temperatures, culture cycles and inoculation amounts were taken to optimize the fermentation conditions. In these experiments, the activity against the bacterium *Bacillus subtilis* and the yeast *Candida albicans* were used to evaluate the effects of the optimization.

**Figure 4:**
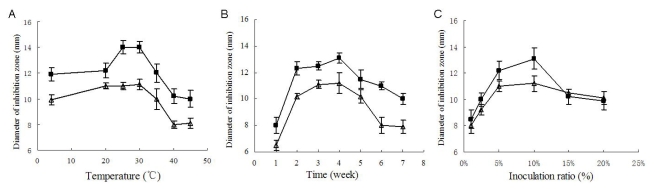
The bioactivity of samples under different culture temperatures (A), under different culture cycles (B), and under different inoculation amounts (C). “▪” presented the activity against *B. subtilis*; “Δ” presented the activity against *C. albicans.*

Temperature and culture cycle are usual key factors of any fermentation. As shown in Figure 4A, the strongest antimicrobial activity was produced when cultivated at 25 °C to 30 °C, and when cultivated at 25 °C, the yield of antimicrobial substances reached its peak value after 4 weeks’ fermentation (Figure 4B).

Inoculum amount was also an important factor. As shown in Figure 4C, when cultivated at 25 °C for four weeks, the culture with 10% inoculation ratio exhibited the strongest inhibition against the two test microorganisms. Inoculation ratios higher than 10% seemed to reduce the bioactive substances’ yield, which was probably due to the exhaustion of nutrition due to the excessively fast growth. On the basis of the above single-factor experiment, a suitable fermentation condition was primarily determined as 4 weeks’ fermentation at 25 °C to 30 °C with an inoculation ratio of 10% (v/v), which was also coincidently close to the fermentation conditions previously used for the antimicrobial assays. As shown in Figure 3, under these conditions (30 days, 10% inoculation ratio, 28 °C) the diameters of inhibition zones against *B. subtilis* and *C. albicans* attained 16.0 ± 0.5 mm and 14.1 ± 0.3 mm, respectively, which were remarkably higher than the peak values in the single-factor experiment.

### 2.5. Preliminary Purification and Characterization of Antimicrobial Compounds

**Figure 5:**
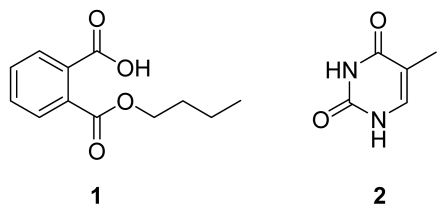
Compounds **1** and **2**.

After a large scale (150 L) anaerobic fermentation under the optimized conditions, an antimicrobial compound **1** (Figure 5) was obtained as a colorless powder by bioguided isolation. The analysis of the ^1^H- and ^13^C-NMR spectra showed that **1** was mono-*n*-butyl phthalate by comparison of its spectral data with data in references [10–11]. Compound **1** showed broad spectrum antimicrobial activities in the assay. The diameters of the inhibition zones against *Candida albicans*, *Staphylococcus epidermidis*, *Escherichia coli*, *Pseudomonas fluorescens*, and *P. aeruginosa* were 7, 7, 7, 6.5, and 7 mm, respectively, at a dose of 50 μg per disc. According to these relatively moderate or weak inhibition zones, compound **1** should be a minor antimicrobial product of SRB-22.

A literature survey indicated that a similar compound, dibutyl phthalate (DBP) has often been isolated from marine plants and microorganisms and there is considerable debate about its origins as a natural product or an environmental pollutant [12–15]. For example, Namikoshi *et al*. reported that DBP from three seaweeds had much higher ^14^C content than the industrial DBP derived from the fossil fuel, i.e., petroleum, which supported a biological origin of the DBP [12]. By a ^14^C-enriched NaHCO_3_ feeding experiment, Chen *et al*. also found that a red alga was able to produce natural DBP. Although mono-*n*-butyl phthalate (MBP) hasn’t been previously reported from marine environment, these data suggest with high probability a possible biological origin for **1**.

**Figure 6:**
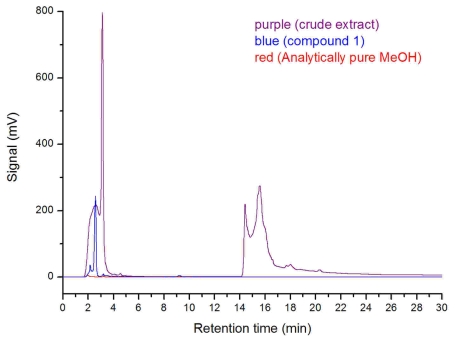
HPLC analysis of compound **1**, crude extract of SRB-22 and analytically pure methanol under the same chromatographic condition.

Additionally, the SRB-22 crude extract for antimicrobial assay was fermented in clean glass serum bottles and dissolved in analytically pure methanol after rotary evaporation. The analytically pure methanol was shown to be DBP and MBP free as tested by high performance liquid chromatography (HPLC), so we conclude that any possible contamination by DBP was avoided. The HPLC analysis of this crude extract also showed the presence of the MBF peak (retention time: 2.34 min; maximum UV absorption at 222 nm and 278 nm) (Figure 6), which further supported the biological origin of **1**. Considering its structure, it may be a biodegraded product of the natural or artificial DBP. As for its exact origin and biosynthesis pathway, further research on ^14^C content measurement or ^14^C-enriched carbon source feeding experiments are needed.

Besides compound **1**, an inactive known compound, thymine (**2**, Figure 5) [16], was isolated and characterized from the *n*-butanol extract of another batch of SRB-22 ferment broth. Its spectral data are not shown.

The other active fractions are still under purification. In the isolation of this anaerobic bacterium’s metabolites, some difficulties have been encountered. For example, some compounds usually showed high similar polarities, insensitivity to chemical chromogenic reagents, or weak UV absorptions, which made the purification inconvenient. However, more efforts and techniques will be put into the isolation of the antimicrobial compounds and further progress is expected.

## 3. Conclusions

By use of convenient agar shake dilution method, a sulfate reducing bacterium SRB-22 was obtained. This isolate was identified as *Desulfovibrio desulfuricans* on the basis of morphology, physiological and biochemical characteristics, and 16S rDNA analysis. In the antimicrobial assay, the extract of isolate SRB-22 showed broad-spectrum activity against two kinds of G^+^ bacteria, three kinds of G^−^ bacteria, and two kinds of fungi. Its mechanism of action is presumed to be different from those of the positive control ampicillin and nystatin. The anti-*Bacillus subtilis* and anti-*Candida albicans* bioautographies showed that at least five antibacterial and three antifungal components were produced by the isolate.

Suitable fermentation conditions for the production of anti-*Bacillus subtilis* and anti-*Candida albicans* compounds was primarily determined to be the cultivation at 25 °C to 30 °C for 4 weeks with 10% inoculation ratio. Under these conditions, the SRB-22 was fermented on a large-scale, extracted and chemically investigated for its antimicrobial compounds. So far, a first active compound, *n*-butyl phthalate (**1**) was obtained and identified and it showed the moderate activity against *C. albicans*, *S. epidermidis*, *E. coli*, *P. fluorescens*, and *P. aeruginosa*.

Sulfate-reducing bacteria, as a big family of chemically underexplored anaerobic microorganisms, are rarely reported as sources of new bioactive secondary metabolites and are expected to be a new resource for novel lead compounds because of their special metabolic pathways [17]. For its broad-spectrum antimicrobial activity, different action modes from ampicillin and nystatin, and potential of producing sorts of active compounds, the isolate SRB-22 is expected to be a fertile producer of novel antibiotics different from the products of the aerobic biosphere. Further research on SRB-22’s bioactive products is ongoing and the results will be reported in due course.

## 4. Experimental

### 4.1. Strain Isolation

The benthal sea water sample was collected using a Nansen bottle in the Heshijiao sea area (121° 30′ 33.95″ E, 38° 48′ 41.78″ N) of Dalian, China, at a depth of about 10 m. The following isolation steps were performed on a common air clean bench: firstly, 50 mL of the sea water sample was inoculated into 450 mL sterile enrichment medium contained in a 500 mL sealed bottle, filled up to the lip. Then the bottle was incubated for enrichment at 30 °C for two weeks. The enrichment medium contained 0.5 g KH_2_PO_4_, 1.0 g NH_4_Cl, 1.0 g Na_2_SO_4_, 2.0 g MgSO_4_·7H_2_O, 5.0 g 70% sodium lactate, 1.0 g yeast extract, 0.1 g CaCl_2_·2H_2_O, 0.5 g FeSO_4_·7H_2_O, 0.1 g sodium thioglycolate and 0.1 g vitamin C in per 1000 mL filtrated sea water and the pH value was adjusted to 7.2–7.4 before autoclaving.

One mL of enriched sample was added to a flask containing 99 mL of sterile sea water and glass beads, and drastically shaken to be a 10^−2^ dilution This 10^−2^ dilution was then further diluted sequentially to give 10^−3^, 10^−4^, 10^−5^, 10^−6^, 10^−7^, and 10^−8^ dilutions in test tubes. A 0.5 mL aliquot of each serial diluton (10^−4^, 10^−5^, 10^−6^, 10^−7^, and 10^−8^) was transferred into a test tube and 10 mL of melted enrichment medium containing 1.5% agar at 50 °C was added [3]. After solidification the tube was sealed with a sterilized overlay consisting of paraffin wax and paraffin oil (v/v, 1/3). The overlay should be over 2 cm thick. Subsequently, the tubes were incubated at 30 °C in an incubator for about 1 week. When black colonies formed in the tube, one selected colony was cut out from the broken agar tube, transferred into a serum bottle full of culture medium, and incubated at 30 °C for about one week. Then, 1 mL of this liquid culture was used for further purification and the above steps were repeated until pure colonies were obtained. The purity was checked by light microscope.

### 4.2. Taxonomy

Routine Gram staining reaction was performed according to the standard method [18]. The cellular size, shape and flagella were observed after phosphotungstic acid negative staining by a transmission electronic microscope (TEM). A desulfoviridin test for the identification of the genus *Desulfovibrio* was performed using the method in the reference [5]. Briefly, 30–40 mL of fresh bacterial suspension was filtered through a 0.45 μm filter membrane, then a drop of 2 mol/L NaOH was added to the membrane and fluorescence under a 365 nm ultraviolet lamp was observed. Red fluorescence was recorded as a positive reaction.

In the carbon source utilization (i.e., electron donor) and growth factor requirement experiments, the carbon source sodium lactate and the growth factor yeast extract in the enrichment medium were removed from the former composition; instead, 0.032 mol/L of different organic carbon sources were added, including sodium lactate, sodium pyruvate, sodium malate, sodium acetate, sodium butyrate, and choline [5]. In some further experiments to study its dependency on sulfate as electron receptor in carbon source utilization, i.e., its ability of fermentative growth, all sulfates in the enrichment medium were also substituted by the corresponding chlorides in addition to the above modification. The above physiological and biochemical experiments were carried out by the following method: the SRB-22 isolate was firstly inoculated into a new serum bottle full of the above corresponding medium with an inoculation ratio of 0.2% (v/v) and cultivated for one week at 30 °C. Then this new culture was used to seed the next generation and this subculture operation was repeated twice. The culture result in the basic medium was taken as negative control. Finally, the growth in the specific medium was recorded and used to evaluate the utilization capability to different carbon sources.

The cells in logarithmic phase were collected by centrifugation and the total genomic DNA was extracted by a standard sodium dodecyl sulfate-proteinase K lysis procedure [19]. The 16S rRNA gene was amplified from the genomic DNA by PCR with the universal primers 27F (5′-AGAGTTTGATCC TGGCTCAG-3′) and 1492R(5′-TACCTTGTTACGACTT-3′) [20]. The PCR products were separated on 0.8% agarose gels. The DNA bands were excised from the gel and purified by using a Qiagen QIA quick gel extraction kit following the manufacturer’s recommended protocol. Both strands of the purified DNA were then sequenced by an automatic DNA sequencer (ABI) with the primers in Takara’s 16S rDNA Bacterial Identification PCR Kit, including RV-M (5′-GAGCGGATAACAATTT CACACAGG-3′), M13-47 (5′-CGCCAGGGTTTTCCCAGTCACGAC-3′), and the seq internal (5′-CAGCAGCCGCGGTAATAC-3′). All sequencing reactions were carried out with an ABI 3700 automated DNA sequencer at the TAKARA Biotechnology (Dalian) Co., Ltd. Finally, the sequence was submitted to GenBank for deposit and BLASTn search.

### 4.3. Sample Preparation for Antimicrobial Assay

Pure bacterial suspension of SRB-22 isolate in logarithmic-phase was used to seed other serum bottles full of enrichment medium for bioassay, with an inoculation ratio of 10% (v/v). After 30 days’ cultivation at 28 °C, 300 mL of the ferment broth was concentrated to dryness *in vacuo* by a rotary evaporator at 45 °C. Then the residue was dissolved in 3 mL of pure methanol and centrifuged at 3,000 rpm for 20 min. The supernatant was used for antimicrobial tests.

### 4.4. Antimicrobial Assay

The antimicrobial tests were performed using the KB paper disk agar diffusion method [21]. The Gram positive bacteria *Staphylococcus epidermidis* ATCC 12228 and *Bacillus subtilis* ATCC 6633, the Gram negative bacteria *Pseudomonas fluorescens* ATCC 13525, *P. aeruginosa* ATCC 9027, and *Escherichia coli* ATCC 11229, and the fungi *Cryptococcus neoformans* ATCC 90112, *Candida albicans* ATCC 10231 and *Rhodotorula rubra* DSM 10403 were used as target microorganisms. After incubation for 24–48 h at 37 °C, the test organisms’ lawns were washed with 0.85% NaCl sterile solution and diluted to 0.5 MacFarland unit (MCF) to be used as standard inoculum suspension. The specific media for test bacteria or fungi were poured into petri dishes and inoculated with 100 μL of the fresh suspension on the surface. Mueller Hinton Agar medium was used for cultivation of the test bacteria, and the Sabouraud Dextrose Agar medium was used for cultivation of test fungi. Each paper disk (φ 6 mm) was added with 100 μL of sample dissolved in methanol and then air dried for use. The extract of the unseeded sterilized enrichment medium was taken as blank control; ampicillin at 6 μg/mL, and nystatin at 100 μg/mL were taken as positive controls; methanol was used as negative control. Finally, the Petri dishes containing the paper disks were incubated for 24 h at 37 °C before the measurement of inhibition zones’ diameters. Each test was performed in triplicate.

### 4.5. Preparative TLC and Bioautography

Firstly, 20 mg of the SRB-22 extract, prepared by the same method described in 4.3, was applied onto a 20 × 20 cm^2^ thin layer chromatography (TLC) plate coated with GF254 silica gel (Qingdao Haiyang Chemical Company, Qingdao, China), developed with a mixture of CH_2_Cl_2_/MeOH/ triethylamine (v/v/v, 10:1:0.02), and air dried. According to the bands under natural daylight and a 254 nm ultraviolet lamp, five fractions 1–5 with increasing polarities were scraped off the TLC plate together with the silica gel powder. Then each fractional samples was ground, eluted from the silica gel with CH_2_Cl_2_/MeOH (v/v, 1:1), concentrated to dryness by rotary evaporator *in vacuo* and finally dissolved in 1 mL of CH_2_Cl_2_/MeOH (v/v, 1:1).

Then 5 μL of each sample was applied on analytical TLC plates (Qingdao Haiyang Chemical Company, GF254 silica gel) with a capillary. The TLC plates were also developed by the same three-phase solvent system and air dried. After 20 min’s sterilization by UV light in air clean bench, the TLC plates were placed upward onto the surface of agar medium in Petri dishes, then 20 mL of melted specific medium at 45 °C (Mueller Hinton Agar for *B. subtilis* and Sabouraud Dextrose Agar for *C. albicans*) containing about 10^6^ CFU/mL of test microorganisms were poured onto the surface of the TLC plates [22]. The Petri dishes were incubated at 37 °C for 16 h for *B. subtilis* and 24 h for *C. albicans*. Then the dishes were stained with methylthiazolyldiphenyl-tetrazolium bromide (MTT) (5 mg/mL) solution for clear observation and photography of inhibition spots.

### 4.6. Study on Fermentation Conditions

To improve the overall yield of the active constituents, temperature, culture cycle and inoculation amount were seperately investigated for their influence on the production of antimicrobial substances.

In the temperature experiment, serum bottles, each filled with 125 mL of enrichment medium, were inoculated with the 10% volume of logarithmic-phase culture and incubated for 4 weeks at 4, 20, 25, 30, 35 and 45 °C, respectively. For each temperature, three replicates were performed. Then the cultures were evaporated to dryness *in vacuo*, dissolved in 1.25 mL methanol and centrifuged to obtain the supernatant for antimicrobial test as described above.

In the culture cycle experiment, the arrangement was similar. The inoculum ratio was 10% (v/v) and the temperature was 25 °C for all. Only the culture cycle was different, i.e., 1, 2, 3, 4, 5, 6 and 7 weeks, respectively.

Likewise, in the inoculation amount experiment, the temperature was 25 °C and the culture cycle was four weeks for all. The difference lay in the inoculation ratios, i.e., 1%, 2.5%, 5%, 7.5%, 10%, 15% and 20% (v/v), respectively.

### 4.7. Large-Scale Fermentation

Three sterilized 50 L plastic barrels containing sterilized enrichment medium were seeded with 7-day old bacterial suspension with an inoculation ratio of 10%. Then the barrels were filled up with culture medium, sealed with airtight caps, and cultivated at room temperature 25~30 °C, for 28 days.

### 4.8. Purification and Characterization of Antimicrobial Compound **1**

The 150 L culture broth was sand filtered to remove FeS precipitate, absorbed by 5 kg of NKA macroporous absorbent resin (The Chemical Plant of Nankai University, Tianjin, China) on a column and consecutively eluted with deionized water and 95% aqueous ethanol. The eluent of the aqueous ethanol was evaporated under reducing pressure, resulting in a residue of 12 g. This extract was subjected to column chromotagraphy (CC) on silica gel (Qingdao Haiyang Chemical Company; 200~300 mesh) eluting stepwise with solvents of increasing polatity from petroleum ether, CH_2_Cl_2_, to MeOH. The CH_2_Cl_2_/MeOH (10:1) fraction (3.1 g) was subsequently subjected to pTLC (GF254 silica gel) developed with CH_2_Cl_2_/MeOH/triethylamine (v/v/v, 10:1:0.02). The eluent (522 mg) from the band with the *Rf* value of 0.2–03 was then separated by CC (RP-18, Merck; MeOH/H_2_O 75:25 to 100% MeOH) and semipreparative HPLC (RP-18, Hypersil, 5 μm, 8.0 × 250 mm; MeOH/H_2_O 85:15, 0.02% triethylamine added; Elite P230 HPLC apparatus, Dalian, China), to yield compound **1** (5.0 mg). The above purification was bioguided using *C. albicans* and *B. subtilis* as indicators. The antimicrobial activities of compound **1** were assayed following the method described in part 4.4, above.

Besides, an inactive compound **2** (4.0 mg) was isolated from the *n*-butanol extract of a previous batch of SRB-22’s ferment broth (60 L), by successive use of CC on silica gel (200~300 mesh), CC on RP-18, and semipreparative HPLC.

The NMR spectra were recorded on a Bruker Avance 500 (500 MHz for ^1^H and 125 MHz for ^13^C) spectrometer using tetramethylsilane (TMS) as internal standard and chemical shifts were recorded as δ values. ^1^H NMR (CDCl_3_) δ_H_: 7.91 (1H, ddd, *J* = 7.1, 1.8, 0.8 Hz, H-3), 7.72 (1H, ddd, *J* = 7.1, 1.8, 0.8 Hz, H-6),7.62–7.55 (2H, m, H-4, 5), 4.34 (2H, t, *J* = 6.7 Hz, H-1′), 1.73 (2H, m, H-2′), 1.45 (2H, m, H-3′), 0.95 (3H, t, *J* = 7.4 Hz, H-4′); ^13^C-NMR (CDCl_3_) δ_C_: 171.0 (C-1, C=O), 168.3 (C-8, C=O), 130.5 (C-2, C), 130.0 (C-3, CH), 131.0 (C-4, CH), 132.0 (C-5, CH), 129.0 (C-6, CH), 133.2 (C-7, C), 66.0 (C-1′, CH_2_), 30.5 (C-2′, CH_2_), 19.2 (C-3′, CH_2_), 13.7 (C-4′, CH_3_).

The HPLC analysis of compound **1** (2.0 mg/mL), crude SRB-22 extract (the same extract used for antimicrobial assays), and analytically pure methanol used for dissolving samples (Kermel Chemical Reagent Company, Tianjin, China) was performed on the HPLC apparatus using an analytical RP-18 column (Hypersil, 5 μm, 4.6 × 250 mm; 0~10 min, MeOH/H_2_O 70:30; 10~11 min, MeOH/H_2_O 70:30~100:0; 11~30min, MeOH). Three μL of each sample was used for analysis.
